# Thiophene-Based Covalent Organic Frameworks: Synthesis, Photophysics and Light-Driven Applications

**DOI:** 10.3390/molecules26247666

**Published:** 2021-12-17

**Authors:** Rubén Caballero, Boiko Cohen, Mario Gutiérrez

**Affiliations:** 1 Departamento de Química Orgánica, Facultad de Ciencias Ambientales y Bioquímica, INAMOL, Universidad de Castilla-La Mancha, Avenida Carlos III s/n, 45071 Toledo, Spain; 2 Departamento de Química Física, Facultad de Ciencias Ambientales y Bioquímica, INAMOL, Universidad de Castilla-La Mancha, Avenida Carlos III s/n, 45071 Toledo, Spain

**Keywords:** thiophene, covalent organic framework, COFs, synthesis, photophysics, catalysis, sensors

## Abstract

Porous crystalline materials, such as covalent organic frameworks (COFs), have emerged as some of the most important materials over the last two decades due to their excellent physicochemical properties such as their large surface area and permanent, accessible porosity. On the other hand, thiophene derivatives are common versatile scaffolds in organic chemistry. Their outstanding electrical properties have boosted their use in different light-driven applications (photocatalysis, organic thin film transistors, photoelectrodes, organic photovoltaics, etc.), attracting much attention in the research community. Despite the great potential of both systems, porous COF materials based on thiophene monomers are scarce due to the inappropriate angle provided by the latter, which hinders its use as the building block of the former. To circumvent this drawback, researchers have engineered a number of thiophene derivatives that can form part of the COFs structure, while keeping their intrinsic properties. Hence, in the present minireview, we will disclose some of the most relevant thiophene-based COFs, highlighting their basic components (building units), spectroscopic properties and potential light-driven applications.

## 1. Introduction

Porous materials have attracted the attention of the research community over the last decades due to their physicochemical properties, such as high surface area along with accessible pores that allow them to encapsulate a wide range of chemical compounds [1,2,3]. As a result, this has boosted their use in a vast number of key modern technologies such as water trapping and splitting, (photo)catalysis, biomedical applications, or optoelectronic devices, to cite few of them [4,5,6,7]. A subclass of porous materials that has attracted much attention are those that present a long-range periodicity (i.e., crystalline materials) such as metal–organic frameworks (MOFs) [8,9,10], hydrogen-bonded organic frameworks (HOFs) [11,12,13], and covalent organic frameworks (COFs) [14,15,16,17,18,19]. While MOFs are porous crystalline materials made of organic linkers attached to metal clusters (secondary building units) through coordination bonds, COFs and HOFs are generally composed of organic linkers, self-assembled through covalent (COFs) or hydrogen bonds (HOFs). In all these materials, the self-assembly (in an ordered manner) of the building blocks enables to predict their final structure, and therefore, to engineer a new library of porous materials with bespoke properties. 

**Figure 1:**
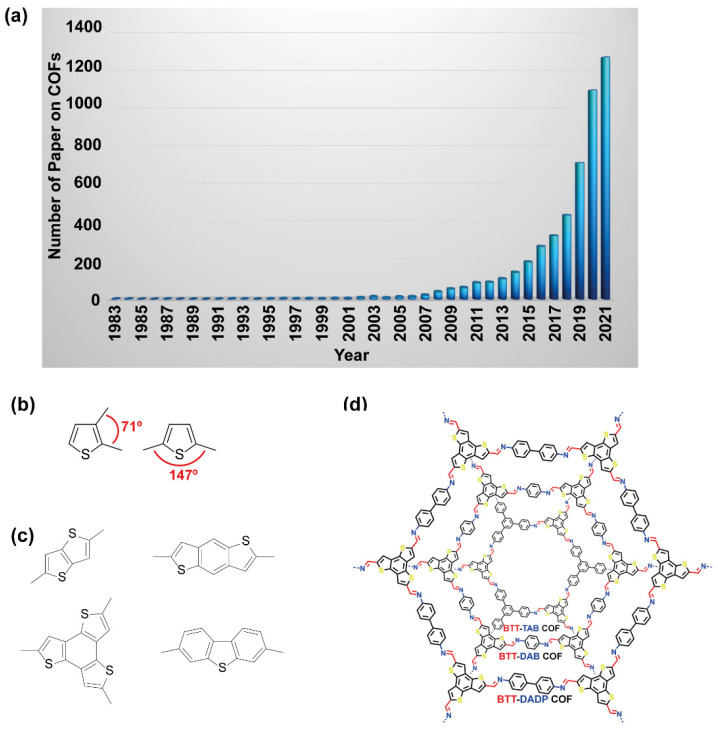
(**a**) Number of peer-review papers on COFs over the last decades. The information was obtained from Scopus. (**b**) Representation of the angles that may form the thiophene-based structures. (**c**) Chemical structure of some representative fused thiophenes. (**d**) Structure of a representative thiophene-based COF. Reproduced with permission from [47]. © 2021 American Chemical Society, Washington, DC, USA.

MOFs, HOFs and COFs share common properties that make them outstanding candidates to be employed in many scientific and technological fields, such as their high surface area, the possibility to control and tune the pore size and distribution, and their chemical flexibility, as the building blocks can be relatively easily replaced by others, leading to a change of their crystalline structure and/or pore size (e.g., isostructural materials) [8,11,14]. However, COFs have some exclusive advantages that cannot be attained by their counterparts (MOFs or HOFs): (a) they lack of toxic metal ions in their structure (contrary tosome types of MOFs or metal-HOFs); (b) they are lighter and have lower density (as they are composed of light elements such as H, C, B, N, O, in contrast to the heavier metals used in MOFs); and (c) higher thermal and chemical resistance in comparison with HOFs due to the elevated strength of the covalent bonds [20,21]. These advantages have driven the field of COFs to a new stage over the last years with an exponential increment in the number of papers published in peer review journals (Figure 1a). Remarkably, the building blocks that form the COFs can be of different nature such as boron-based, imine-based or triazine-based, among others [22,23]. However, a new type of COFs based on thiophene building blocks has gained much attention owing to the promising characteristics (e.g., conductivity, photocatalytic activity) of the thiophene derivatives that can be retained when self-assembled in a COF structure [24,25].

Thiophene derivatives are widely used scaffolds in organic chemistry [26]. They are present in numerous natural as well as synthesized compounds with a wide range of applications in biological and molecular materials fields [27,28,29,30,31]. Thiophene is a stable π-aromatic, five-membered ring compound, with a very well-known chemistry; its aromaticity allows substitution in each of the alfa or beta positions that can be easily controlled under mild reaction conditions. However, the thiophenechemistry is still a matter of interest as some established substitution methods for chemical derivatization are being modified and improved, while novel methods implying sustainable synthetic approaches are being developed [32]. The most common and straightforward scaffold modification is the introduction of electroactive or solubilizing groups in positions 2–5 [33,34]. Some more recent methodologies involve the fusion with other aromatic rings yielding large aromatic π surfaces [35], or even changes to their oxidation state by the oxidation of the sulfur atom or formation of quinoidal structures [36]. All the modifications can thus influence the thiophene planarity and the electron density of the final products. Hence, a judicious selection of the synthetic substitution strategy may enable the design of a wide assortment of thiophene derivatives with bespoke physicochemical properties, and therefore, allowing to engineer a vast number of new thiophene-based molecules and materials with potential applicability in many fields of science and technology [37,38].

Regardless of the outstanding properties of the thiophene derivatives, the use of these linkers in the construction of COFs is still in its infancy, so there is a substantial margin to explore in this field. In general, most of MOF, COF and HOF materials are constructed through building blocks that provide angles of 60°, 120° and/or 180°, while the thiophene, having five-member rings, cannot provide a factor angle of 360°, hampering the formation of crystalline materials (Figure 1b). Despite of this drawback, COFs based on thiophenes have been reported [24,39,40]. Although few examples are available where thiophene monomer forms part of the building blocks, the construction of crystalline 2D assemblies can be more easily achieved by using building units based on fused thiophene with other rings (Figure 1c) [41]. This is the example of thieno[3,2-*b*]thiophene [42,43,44,45,46], trithieno[*b*]benzene (Figure 1d) [47], dithieno[1,2,4,5-*b*,*b*]benzene [48,49,50], or dibenzo[*b*,*d*]thiophene [51], where angles of 120°, 180°, or close, are formed. This review intends to give a comprehensive view from the synthesis of these COF materials to their photophysical properties and potential light-driven applications. To this aim, we will firstly focus on COFs constructed with thiophene molecules as the building blocks, to continue with COFs made of oligothiophenes and functionalized thiophenes, finalizing with thiophene-based COFs as a matrix for the encapsulation of guests.

## 2. Thiophene Monomers as Building Units

**Figure 2:**
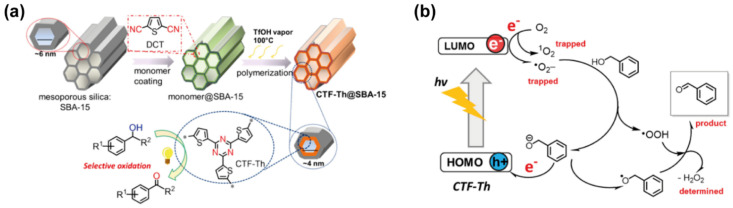
(**a**) Schematics of the synthetic steps followed for the fabrication of the thiophene-based COF (CFT-Th) using SBA-15 as a mesoporous nanoreactor. The scheme also includes the structure of the thiophene-triazine building block. (**b**) Representation of the proposed photocatalytic mechanism of the selective oxidation of alcohols by the photoexcited CFT-Th material. Reproduced with permission from [52]. © 2021 American Chemical Society.

Until now, and to the best of our knowledge, there are limited examples of COFs fabricated with thiophene monomers as the building block unit. Indeed, the closest COF made of thiophene monomers that can be found in the literature is a thiophene-based covalent triazine framework (CFT), where the monomer unit is a thiophene joined to a triazine moiety (Figure 2a) [52]. In the first reported example, the thiophene-COF (CTF-Th) was synthesized onto a mesoporous silica SBA-15 material (Figure 2a), via cyclization polymerization of the building block unit (2,5-dicyanothiophene) in the presence of vapors of trifluoromethanesulfonic acid (TfOH). The photophysical characterization of this material showed a broad absorption from 250 to 520 nm and an emission band with the maximum centered at 530 nm. The band gap was estimated to be 2.47 eV, while the lowest unoccupied molecular orbital (LUMO) was −0.72 V (obtained through cyclic voltammetry). This hybrid material was used to photocatalyze the oxidation of benzyl alcohol under blue light irradiation, showing conversion efficiencies and selectivity over 99%. The authors studied the mechanism and proposed that upon irradiation, CTF-Th photogenerates electrons, which produce activated forms of oxygen (•O_2_^−^ and ^1^O_2_). These activated species extract a proton of the benzyl alcohol, giving place to the formation of its anionic form by obtaining •OOH species. Then, the benzyl anion is oxidized by the photogenerated holes into an anionic radical, and subsequently oxidized by the •OOH species yielding the benzaldehyde as the final product (Figure 2b).

**Figure 3:**
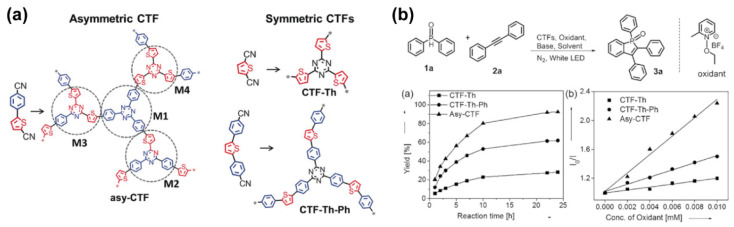
(**a**) Chemical structures of the asymmetric and symmetric thiophene-based CFTs. (**b**) The upper part represents de reaction catalyzed by the thiophene CFT. The right panel graph on the bottom represents the yield (in %) of the reaction catalyzed by the asymmetric and symmetric thiophene-CFTs, respectively, while the left panel graph on the bottom is the representation of the Stern-Volmer analysis obtained by the quenching measurements of CFTs and oxidants in DMF. Reproduced and adapted with permission from [53]. © 2021 Wiley-VCH Verlag GmbH and Co. KGaA, Weinheim, Germany.

In a consecutive work, the authors reported on the synthesis of an asymmetric thiophene-based CFT and two symmetric thiophene-CFTs (Figure 3a), and then compared their photocatalytic activities [53]. The synthesis of the asymmetric and symmetric COFs was performed following the same procedure, which involved a first step where the corresponding building block monomer and SiO_2_ nanoparticles were dispersed in tetrahydrofuran (THF) in an ultrasonic bath for 30 min. Then, the solvent was evaporated under vacuum, and the solid was exposed to vapors of TfOH in a degassed environment. The TfOH induced a trimerization of 5-(4-cyanophenyl)thiophene-2-carbonitrile in the solid state. Finally, the sample was washed with DCM in a Soxhlet extractor, and the silica nanoparticles were removed with NH_4_HF_2_ (4M). Afterwards the authors explored the photophysical properties of these COFs and found that: (i) all the COFs exhibited a broad absorption spanning from 250 to 800 nm, with an estimated band gap of 2.48 and 2.42 eV for the symmetric COFs and 2.3 eV for the asymmetric one; (ii) the cyclic voltammetry measurements provided the values of the LUMO of the asymmetric COF (−1.30 V), which was slightly higher than that of the asymmetric counterparts (−1.24 and −1.02 V, respectively); (iii) the emission lifetime of the asymmetric COF was shorter (1.27 ns) than those obtained for the symmetric ones (1.66 and 1.57 ns), which the authors attributed to a possible enhancement of the delocalization of the excitons in the asymmetric COF; and (iv) the photocurrent measurements demonstrated that the asymmetric COF showed an improved photocurrents compared to its symmetric equivalents. All these results suggested that the asymmetric COF would function as a photocatalyst better than the symmetric ones, and to prove this, the authors explored the formation of 1,2,3-triphenylphosphindole 1-oxide from diphenyl-phosphine oxide and diphenylacetylene, using the COFs as photocatalysts and irradiating the reaction with visible light. It was demonstrated that the conversion yield was 93% when the asymmetric COF was used as photocatalyst. This value was consistently higher than that of the conversion rate obtained for the symmetric COFs (22 and 63 %), and therefore, confirming the better photocatalytic aptitudes of the asymmetric thiophene-COF (Figure 3b).

Despite of these promising examples, the inappropriate angle of the five-membered ring thiophene molecules hinders their use as fundamental monomer units in the construction of COFs. To circumvent this handicap, researchers have established elegant alternative avenues, where the use of oligothiophenes or functionalized-thiophene molecules serving as the building blocks have opened a new approach for obtaining thiophene-COFs. In the following sections, we will therefore overview some of the most representative COFs based on thiophene derivatives, covering from oligothiophenes, functionalized thiophenes, and some exciting examples of hybrid guest@thiophene-COF materials.

## 3. Oligothiophenes as Building Units

Oligothiophenes are considered as a promising alternative to smaller thiophene building units in the construction of supramolecular materials. They are large and conjugated structures, with a characteristic π-π* transition that generally produces a red shift of their absorption spectra. Oligothiophenes and their derivatives have shown excellent performance in many scientific and technological fields (biosensors, OLEDs, organic photovoltaics, among others) [30,54,55], boosting their development and research interest.

**Figure 4:**
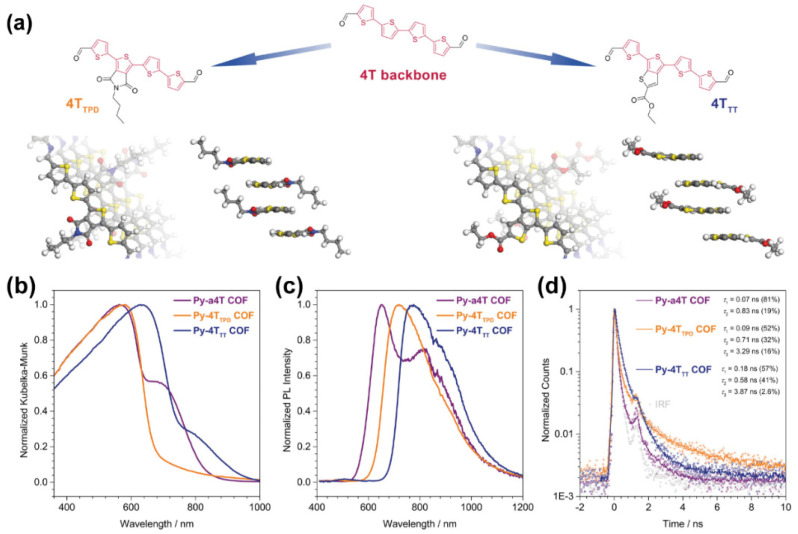
(**a**) Schematic representation of the asymmetric approach yielding quaterthiophene-based donor–acceptor building blocks, where one thiophene backbone can be replaced by an electron-deficient extended thiophene. (**b**–**d**) Normalized (**b**) diffuse reflectance converted to Kubelka-Munk (K-M), (**c**) emission spectra, and (**d**) ps-ns emission decays of Py-a4T (purple), Py-4T_TDP_ (orange) and Py-4T_TT_ (blue) COFs. Reproduced and adapted with permission from [40]; © 2021 American Chemical Society. Further permissions related to the material should be directed to the ACS.

In the field of COFs, oligothiophenes have demonstrated their ability to form planar and stackable 2D structures, piling up to ordered porous frameworks [40], being the latter different from standard 3D COFs, which are supramolecular structures with covalent bonds extending in the 3 dimensions. For example, Bein and coworkers applied a novel concept in which an asymmetric functionalization of the oligothiophene enabled to surpass common drawbacks frequently found during the growth of COFs, such as the very low solubility of extended building blocks, or the impediment of an appropriate face-on oriented packing of the fundamental units required for the construction of COFs [40]. This asymmetric functionalization strategy allowed to incorporate alkyl chains in the building blocks, increasing their solubility while keeping a suitable distance among the oligothiophene layers. Furthermore, electronic modifications of the backbone were also incorporated via the addition of thiophene-based acceptor units, such as 4H thieno[3,4-*c*]pyrrole-4-,6(5H)-dione and thieno[3,4-*b*]thiophene (Figure 4a) [40]. Combining these modified 4T building blocks with pyrene-based moieties resulted in a series of highly crystalline quaterthiophene-linked COFs. This strategy also promoted a facile modification of the electronic properties of the 4T backbone via incorporation of electron-deficient subunits, thus forming donor−acceptor type molecules. The spectroscopic and time-resolved photodynamics of these COFs shed some light about their promising potential in photonic applications (Figure 4b–d). The absorption of these COFs falls in the whole visible spectral region (from below to 400 nm to 800 nm, Figure 4b), while they emit light in the reddest part of the visible spectrum (from 654 to 773 nm, Figure 4c). Additionally, the photodynamics of these COFs further suggests the occurrence of a photoinduced charge transfer process, which is the basis of many optoelectronic applications [40]. While the decay curves of the building blocks are monoexponential with lifetimes ranging from 0.5 to 2 ns, those of the COFs exhibited a fastest decay with 50% of the photons decaying within 100–200 ps (Figure 4d). This was attributed to a competition between the radiative deactivation of these COFs and the charge transfer pathway.

## 4. Functionalized Thiophenes as Building Blocks

Another promising alternative for the construction of thiophene-based COFs is the use of functionalized thiophenes as the building constituents. This section will be the core of the review as up until now, most of the thiophene-based COFs are constructed by employing functionalized thiophenes as building units.

**Figure 5:**
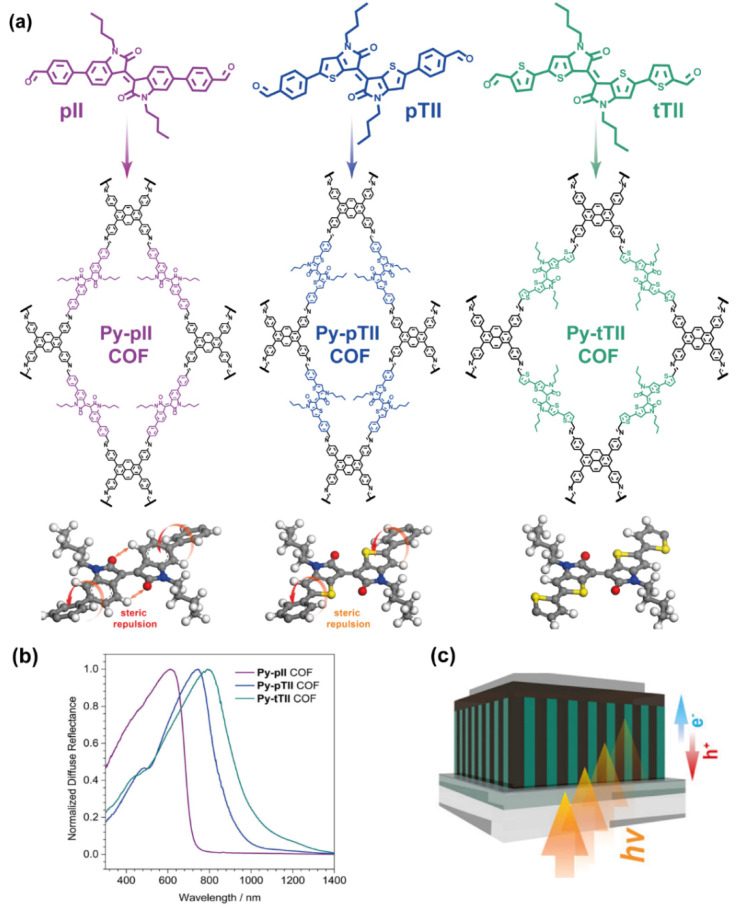
(**a**) Schematic representation of the pyrene-thieno-isoindigo based COFs, showing the steric repulsion for the pII and pTII COF structures that causes the rotation of the phenyls groups, and the planar tTII COF structure. (**b**) Absorption spectra of Py-pII, Py-pTII and tTII COFs. (**c**) Schematic representation of the photodetector made using the pyrene-thieno-isoindigo based COFs. Reproduced and adapted with permission from [56]; © 2021 American Chemical Society. Further permissions related to the material should be directed to the ACS.

In a recent study, thiophene was used to modify the core of *N*,*N′*-dimethyl-isoindigo to fabricate a thiophene-based COF [56]. This isoindigo derivative has shown that due to the steric repulsion between the protons of the benzene ring and the oxygen atoms of the ketopyrrole, it crystallizes in a slightly twisted configuration with a rotation of the two oxindole rings along the central double bond [57]. For COFs, this deviation from a truly planar conformation might have several negative connotations. It might not only reduce the effective π-conjugation in the molecule but could also affect the crystallinity and stability of the framework. In the thieno-modified isoindigo core (TII) the unfavourable repulsion between the ketones and the adjacent hydrogen atoms is replaced by an electrostatic attraction between the sufficiently spaced ketones and sulphur atoms, thus rendering the molecule entirely planar [58,59]. Bessinger et al. applied these concepts to test the influence of this added planarity on the structure and electronic properties of COFs based on the 5,5′-bis(4-formylphenyl)-*N*,*N′-*dibutyl-thienoisoindigo (pTII), which features a planar core that is flanked by slightly tilted phenyl rings. Replacing the latter by thiophenes resulted in a planar conformation of the 5,5′-bis(2-formylthiophen-5-yl)-*N*,*N′-*dibutyl-thienoisoindigo (Py-tTII) building block (Figure 5a) [56]. The integration of the isoindigo- and thienoisoindigo-containing building blocks in the COFs produced strongly coloured frameworks that absorb light across the visible and parts of the NIR spectrum (Figure 5b). The 2D COFs possess very anisotropic electronic properties with the highest conductivity typically being along the π-stacked molecular columns. For application as an active component in a photodetector device, these columns should therefore be aligned vertically to the substrate [60]. The growth of oriented films on non-epitaxial substrates, during which the anisotropy of the COF structure generates the preferred vertical orientation, as it was previously reported for boronate ester-linked COFs [61].

Therefore, the authors used this previously developed method for the growth of the Py-tTII COF on an ITO/MoO_x_ (indium−tin oxide/molybdenum oxide) transparent electrodes (Figure 5c) [56]. Growing these materials as an oriented thin film allowed the construction of an inter-digitated heterojunction upon infiltration of the COF pores with a soluble fullerene derivative ([6,6]-phenyl C_71_ butyric acid methyl ester, PC_71_BM). While the absorption of the COF is enough to provide absorption up to 750 nm, the incorporation of PC_71_BM, a fullerene derivative, broaden the light absorption, spanning until ~1100 nm. This heterojunction was successfully applied as the active layer in a UV- to NIR-responsive photodetector. Moreover, it was shown that the spectral response of the device could be switched reversibly from blue- and red-sensitive to green- and NIR-sensitive by changing the bias voltage.

**Figure 6:**
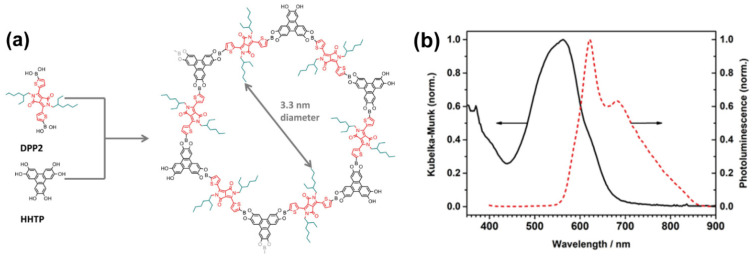
(**a**) Schematic representation of the building blocks, DPP2 and HHTP, used for the fabrication of the DPP2-HHTP-COF, along with a representation of the chemical structure of the latter. (**b**) Normalized diffuse reflectance (transformed to K-M) and emission spectra of DPP2-HHTP-COF in the solid state. Reproduced and adapted with permission from [62]; © 2021 American Chemical Society. Further permissions related to the material should be directed to the ACS.

Diketopyrrolopyrrole (DPP), is another dye based on thiophene that has been used in the construction of a COF (DPP2-HHTP-COF, where DPP2 is diketopyrrolopyrrole, and HHTP is hexahydroxytriphenylene), linked to triphenylene by boronic acid esters (Figure 6a) [62]. For other applications such as organic semiconductors or dyes in organic photovoltaics, DPP is a bulky chromophore and eventually can hamper the totally eclipsed stack of the 2D polymers, yielding J-aggregates in the formation of the final material [63,64]. Nonetheless, in this case, X-ray diffraction of the DPP2-HHTP-COF material in powder state gave well-defined peaks, reflecting its highly crystallinity. DPP was chosen due to its electron acceptor ability and broad absorption, covering a wide range of wavelengths from the UV (<350 nm) up to ~700 nm [62]. The formed material has demonstrated outstanding properties of light absorption (<350 nm–700 nm, Figure 6b), light emission (two peaks at 620 and 680 nm, Figure 6b) and conduction. Its conducting properties were investigated on compressed pellets of the crystalline material, showing conductivity values of up to 2.2 × 10^−6^ S cm^−1^_._ In addition to this, the authors also synthesized a polymer non-crystalline material using the same raw reactants (DPP2 and HHTP), but changing the synthetic conditions, and found that the conductivity of the latter decreased one order of magnitude, giving a value of 2 × 10^−7^ S cm^−1^. The outstanding optical properties (broad absorption) together with its high conductivity, reflect the huge potential of this material for being employed as active layer in solar cells or transistor devices, opening an exciting pathway for the development of new COF materials based on DPP.

**Figure 7:**
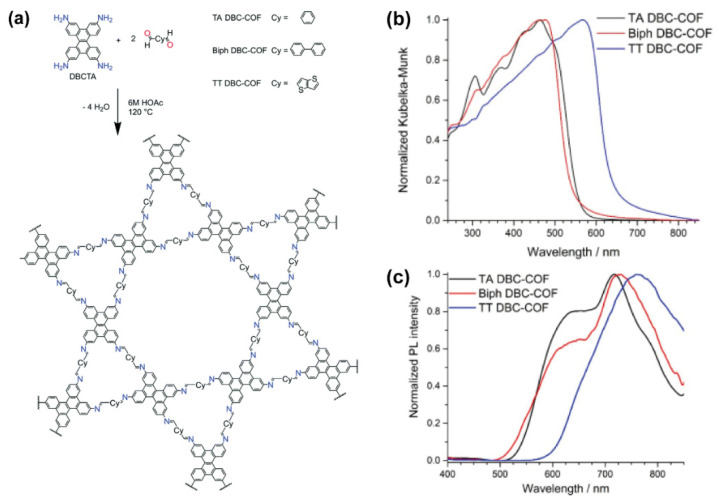
(**a**) Chemical structures of the building blocks used for the synthesis of the DBC-based COFs, together with the chemical structure of the latter. (**b**) Absorption and (**c**) emission spectra of the TA DBC-, Biph DBC-, and TT DBC-COFs. Reproduced and adapted with permission from [65]. © Copyright 2019 Royal Society of Chemistry, London, UK.

In another example, the combination of dibenzo[g,p]chrysene (DBC) with thieno[3,2-b]thiophene (TT) linked by imine groups produces a stackable COF, named TT DBC-COF, with dual triangular and hexagonal 2D cavities (Figure 7a) [65]. In addition to that COF, the authors also synthesized two other COFs in which the TT linker was replaced by terephthalaldehyde (TA) and biphenyl (Biph) building blocks. The obtained COFs combine a conjugation in the a,b-plane with a tight packing of adjacent layers (about 3.6 Å interlayer distances) guided through the molecular dibenzo[*g*,*p*]chrysene node serving as specific docking site for successive layers (the 2D COFs are piled up by π-π stacking to form an ensemble material) [65]. The resulting COFs exhibit a hexagonal dual pore kagome geometry. Interestingly, in comparison to the individual emission of the molecular components, the photoluminescence maxima of all the three DBC-COFs were significantly red-shifted, which suggested the emergence of new electronic structures through the formation of the frameworks. The stacked COFs were predicted to yield smaller band gaps than the isolated COF sheets, which was confirmed by the experimental observations. The authors explained this result in terms of the better π-orbital overlap between the stacked chrysene docking sites due to lower dihedral angles, resulting in short π-distances. Using TT, instead of TA or Biph scaffolds, provides morphological benefits such as tighter π-π stacking, which in turn affects its photophysical properties. More specifically, TT DBC-COF is a dark red solid with broad UV–visible absorption spectrum, and with a small HOMO-LUMO band gap (2.00 eV in comparison to 2.38 and 2.30 eV for TA DBC-COF and Biph DBC-COF, Figure 7b). Additionally, its photoluminescence spectrum is broader and red shifted (up to 760 nm) with respect to the other two COFs (Figure 7c). These promising photophysical properties, along with the capacity to encapsulate guests in the interior channels, indicate that the DBC-COFs in general, and TT DBC-COF in particular, are potential materials for implementation in optical devices.

**Figure 8:**
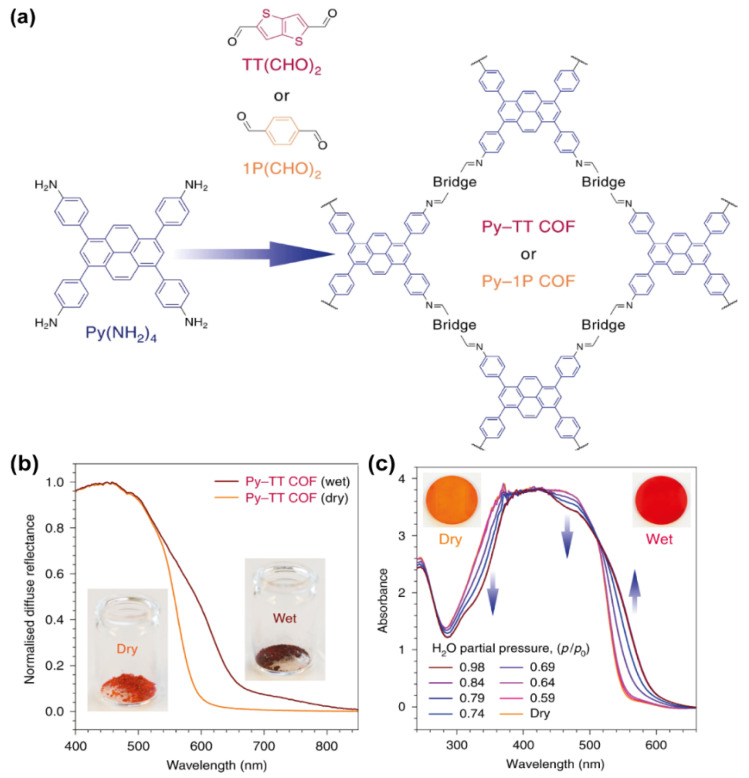
(**a**) Representation of the chemical composition of the building units and the corresponding Py-TT and Py-1P COFs. (**b**,**c**) Absorption spectra of Py-TT COF before and after its interaction with different pressures of water in the form or (**b**) powder and (**c**) film. The inset are real photos where the change in colour after its exposure to water can be followed even by the naked eye. Reproduced and adapted with permission from [66]. Copyright © 2021 Springer Nature, Basingstoke, UK.

Thiophene-based COFs have been also proposed as solvatochromic sensors [66]. The COF structure may allow the encapsulation of solvent molecules via the pores of the framework, thus influencing the properties of the material. Combinations of electron-rich pyrene derivative (1,3,6,8-tetrakis(4- aminophenyl)pyrene, Py(NH_2_)_4_) with more electron-deficient aldehyde counterparts can produce electronic transitions with a varying degree of charge-transfer character across the conjugated imine bond. A solvatochromic COF (Py–TT COF, Figure 8a) based on thiophene (thieno-[3,2-b] thiophene-2,5-dicarboxaldehyde, TT(CHO)_2_) has shown strong and fast solvatochromic response both in bulk solid and grown as a film on a non-epitaxial substrate [66]. Its solvatochromism has been compared with other pyrene-based COFs with decreasingly electron-deficient aldehyde counterparts: (1) a linear acene dialdehyde, 1P(CHO)_2_ that increases the polarity within the linear bridge and doubles the number of weakly accepting imines with a much stronger charge-transfer character; and (2) tetradentate 1,3,6,8-tetrakis(4-formylphenyl)pyrene, Py(CHO)_4_, which produces the smallest donor–acceptor contrast, derived mainly from the slightly polarized, electron-accepting imine. Among the 3 pyrene-based COFs, the Py-TT one showed the best solvatochromic response. When Py–TT COF was exposed to a humid atmosphere, a red shift in the absorption (Figure 8b,c) as well as in the PL was observed, indicating a stabilization of the excited state by the pore medium. This was accompanied by a quenching of the PL by more than 95% compared to the dry material, suggesting that the increased dielectric screening due to the water molecules helps to overcome the Coulomb barrier and sustain a more charge-separated state [67,68]. Based on both the experimental data and theoretical calculations, the authors concluded that the solvatochromism is of purely electronic origin and does not involve structural or chemical changes in the framework, suggesting that in these materials the electronic transitions can be manipulated reversibly, and that intramolecular charge transfer can be facilitated via the inclusion of chemically inert guest molecules, thus favouring the development of stimuli-responsive organic electronics.

**Figure 9:**
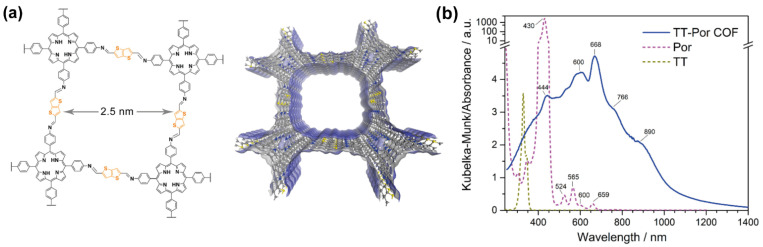
(**a**) Chemical composition and schematic representation of the crystalline structure of TT-Por COF. (**b**) Diffuse reflectance (transformed to K-M) spectra of the thieno[3,2-*b*]thiophene and porphyrin linkers compared to the one of the TT-Por COF. Reproduced and adapted with permission from [69]; © 2021 American Chemical Society. Further permissions related to the material should be directed to the ACS.

Thieno[3,2-*b*]thiophene was also combined with porphyrins, another π-planar and a strong chromophore, to form a thiophene-porphyrin based COF, TT-Por COF (Figure 9a) [69]. The condensation of the amino derivates of the porphyrin and the bisaldehyde derived of the thieno[3,2-*b*]thiophene gave an imine group, that enhanced the p-conjugation and hence induced a strong absorption of light. The resulting 2D lattice is singular, formed of square holes instead of hexagonal or triangular ones (Figure 9a). In the assemble of the stacked structure, the porphyrins pile up giving rise to J-aggregates, as demonstrated by the X-Ray diffraction patterns, TEM microscopy and theoretical calculations. In comparison with the building units, the TT-Por COF material showed an enhanced absorption spanning from the UV (<350 nm) to the NIR (1200 nm) as a consequence of the presence of the J-aggregates (Figure 9b). It should be noted that the authors did not take into consideration the metalation of the porphyrin, which is expected to influence the J-aggregation grade, and thus, the photophysical properties of the molecule.

**Figure 10:**
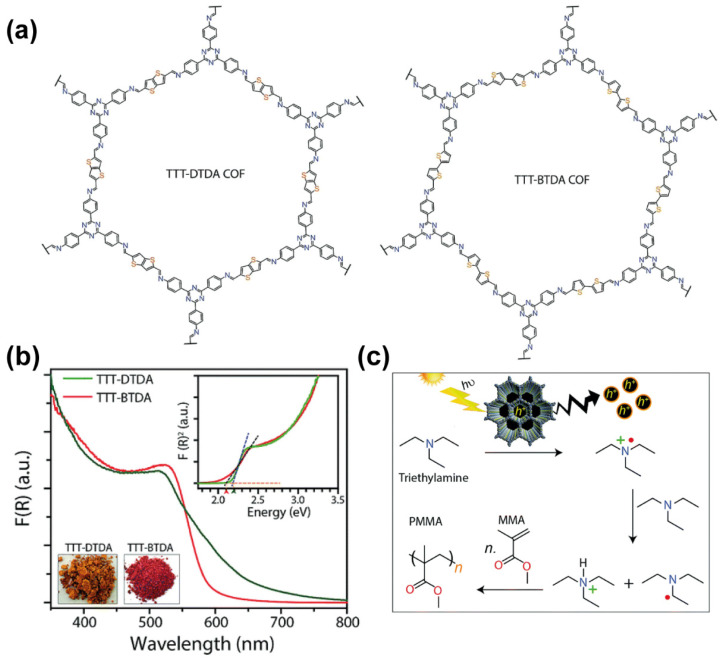
(**a**) Chemical structures of the TTT-DTDA and TTT-BDTA COFs. (**b**) UV-Vis diffuse reflectance spectra of TTT-DTDA and TTT-BDTA COFs. The insets are real photos of the COFs powder and a representation of the diffuse reflectance vs. the energy (eV) used to estimate the band gap of the materials. (**c**) Proposed mechanism for the photopolymerization of MMA to yield PMMA. Reproduced and adapted with permission from [70]. © 2021 Royal Chemical Society.

In another example, two electron-rich thiophene derivatives (thieno[3,2-*b*]thiophene-2,5-dicarbaldehyde and [2,2′-bithiophene]-5,5′-dicarbaldehyde) were combined with an electron-deficient 4,4′,4′′-(1,3,5-triazine-2,4,6-triyl)trianiline to yield two types of donor-acceptor COFs named as TTT-DTDA-COF and TTT-BTDA-COF (Figure 10a) [70]. The PXRD patterns of both COFs showed a strong reflection peak at 2θ = 2.68 and 2.52 degrees, respectively, which was assigned to the (100) facet of a primitive hexagonal facet. In addition to that, a broad reflection signal at around 25 degrees was attributed to the (001) facet, indicating the formation of 2D-COFs with a π-π stacking distribution. Interestingly, the absorption spectra of both COFs are very broad covering from the UV up to 600 nm for TTT-BDTA and 800 nm for TTT-DTDA COFs (Figure 10b). In addition to that, EPR measurements proved that under photocatalytic conditions (i.e., irradiating with light), both COFs were able to generate an electron-hole pair, which is the fundamental for any photocatalytic reaction. Encouraged by that, the authors used both COFs to photopolymerize methyl methacrylate (MMA) to yield polymethyl methacrylate (PMMA) following the mechanism described in Figure 10c, and with conversion efficiencies of 63% (TTT-DTDA) and 54 % (TTT-BTDA).

**Figure 11:**
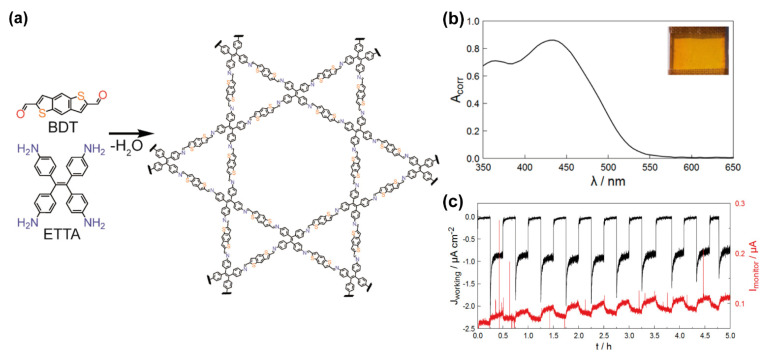
(**a**) Chemical structure of the building units and the BDT-ETTA COF. (**b**) Absorption spectrum of BDT-ETTA COF deposited on a ITO transparent substrate. The inset is a real photo of the BDT-ETTA COF film. (**c**) Graph representing the chronoamperometric data of a BDT-ETTA film recorded at 0.4 V vs. reversible hydrogen electrode (RHE) under chopped AM 1.5 illumination. Reproduced and adapted with permission from [71]. Copyright © 2021, Nature Publishing Group, Berlin, Germany.

Thiophene-based COFs have been also proposed as photoelectrodes for light-driven water electrolysis for the generation of hydrogen as a sustainable way of energy production [49]. For instance, T. Sick et al. described the synthesis of a novel COF (BDT-ETTA COF, Figure 11a), where the building units were a 4-fold amine-functionalized tetraphenylethylene (1,1′,2,2′-tetra-p-aminophenylethylene, ETTA) and a linear dialdehyde benzo[1,2-*b*:4,5-*b*′]dithiophene-2,6-dicarboxaldehyde (BDT). For using this COF as electrode in a photoelectrochemical cell, the authors deposited it onto a transparent fluorine-doped tin oxide (FTO) and indium tin oxide (ITO) substrates. Interestingly, as a fully conjugated and rich in electronic density, BDT-ETTA COF presents a broad absorption ranging from the UV (<350 nm) to the visible range (up to 550 nm, Figure 11b) and a p-type semiconductor behaviour. The piled-up structure is fully stacked leading to tubular and uniform pores in the z-axis. As a differential feature with other materials presented in this minireview, the dibenzothiophene scaffold comes to enhance the planarity of the 2D structure distorted by the less planar tetraphenyl ethylene as demonstrated by the X-ray diffraction pattern where the (100) peak appears at a larger angle than the expected [71]. Using the same technique in combination with TEM microscopy, the authors studied the crystallinity of the obtained material. The solid presented a high stability in several media and in a wide pH range (from 1 to 13). Its best performance was observed when the COF was grown over a conductive surface (like ITO covered glass), giving rise to a highly crystalline surface. The crystalline material has a LUMO energy level with a value that is higher than the H_2_O/H_2_ redox pair in solution over the entire pH range. If the material is deposited over a transparent substrate, its electronical configuration enables hydrogen production by photocatalytic water splitting (Figure 11c).

**Figure 12:**
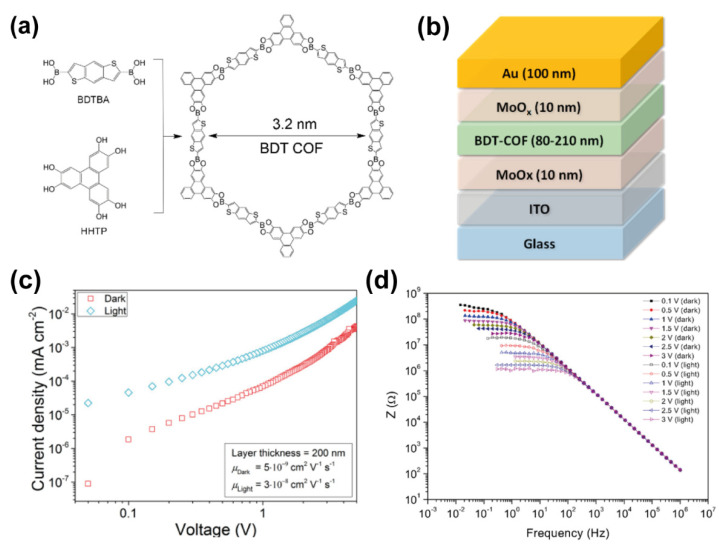
(**a**) Representation of the chemical structures of the BDTBA, HHTP linkers and BDT COF. (**b**) Schematic representation of the architecture of the BDT-COF based HOD. (**c**,**d**) Graphs showing the (**c**) current density vs. voltage and (**d**) impedance of the BDT-COF based HOD in the dark and under illumination. Reproduced and adapted with permission from [72]; © 2021 American Chemical Society. Further permissions related to the material should be directed to the ACS.

Oriented thin films of COFs are also an interesting approach for triggering charge-carrier transport, which is the keystone of many advanced optoelectronic applications. In this sense, D.D. Medina et al. demonstrated that it is possible to fabricate oriented thiophene-COF thin films to investigate the charge-carrier migration happening in this material [72]. In this work, the authors grown the oriented BDT-COF thin films by a solvothermal condensation of benzo[1,2-*b*:4,5-*b*]dithiophene-2,6-diyldiboronic acid (BDTBA), and the polyol 2,3,6,7,10,11-hexahydroxytriphenylene (HHTP) as shown in Figure 12a [72]. The crystallinity of the material was assessed both experimentally (by XRD, TEM and AFM) and theoretically. Thin films were synthesized in an oriented fashion on different conductive substrates and the directional charge carrier transport along the COF molecular columns and in the COF planes, along with the capacitance, recombination resistance and dielectric constant were studied by constructing hole-only devices (HODs, Figure 12b). The study demonstrated a hole mobility along the COF layers and that the charge transport depends on the COF film thickness. This dependency can be attributed to transport barriers within the BDT-COF layers that cannot be easily overcome through alternative transport paths. Moreover, the authors performed light-dependence hole mobility measurements on the HODs under illumination and found a 3-fold increase in the hole mobility (from 5 × 10^−9^ cm^2^ V^−1^ s^−1^ in dark to 3 × 10^−8^ cm^2^ V^−1^ s^−1^ upon illumination, Figure 12c). The photoactivity of the BDT-COF HODs was also demonstrated by impedance experiments comparing the device under illumination and in the dark (Figure 12d). The authors also reported a low dielectric constant for the COF films, assigned to the highly porous character of the COF. Simulations have shown a certain degree of layer-to-layer displacement due to the highly polarized B−O bonds, which leads to a decrease in the layer-to-layer electronic coupling, giving rise to a narrowed density of states at the valence band edge.

**Figure 13:**
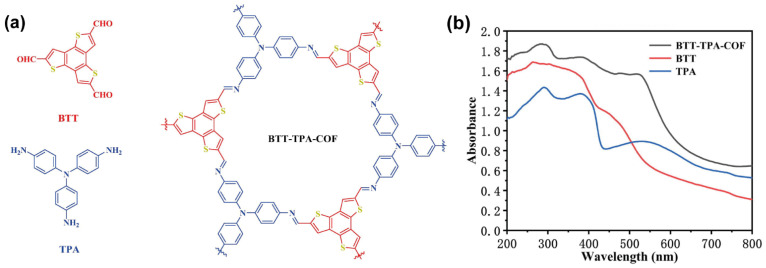
(**a**) Chemical structures and (**b**) absorption spectra of the BTT and TPA linkers, and the BTT-TPA-COF. Reproduced and adapted with permission from [39]. © 2021 Elsevier Inc., Amsterdam, The Netherlands.

Thiophene derivatives can be also used as building blocks to generate angles of 120° for polymerization in two dimensions. One example is the benzo[1,2-*b*:3,4-b′:5,6-*b*′′]trithiophene, also known as benzotrithiophene or BTT. This scaffold has C3 symmetry, a rigid and planar structure and, as a large fused aromatic system, can behave as electron acceptor moiety. B. Luo et al. used this BTT unit in combination with triphenylamine TPA linker to yield the BTT-TPA-COF (Figure 13a), in which the TPA behaves as the electron donor of the coupling pair [39]. In this material, the piled structure corresponds to a totally eclipsed fashion where each BTT is located between two others. This strategy allows to easily adjust the band gap, energy level and photoelectric performance of the framework. The BTT-TPA-COF has a narrow band gap with strong absorption in the spectral range between 200 nm to the near infrared (up to 800 nm, Figure 13b) [39]. Besides, the material is stable in a broad temperature (up to 300 °C) and pH (from 1 to 13) ranges. This COF has large porosity that allows the inclusion of small molecules. More interestingly, this COF showed a successful photocatalytic activity in the conversion of o-phenylenediamine to 2- arylbenzimidazole reaching efficiencies of up to 94 and 97% in methanol and ethanol, respectively. In addition to that, the photocatalytic reaction using BTT-TPA-COF was very reproducible after eight cycles, pointing out its outstanding photocatalytic performance and reusability.

## 5. Thiophene-Based COFs Incorporating Guests in Their Porous Structure

**Figure 14:**
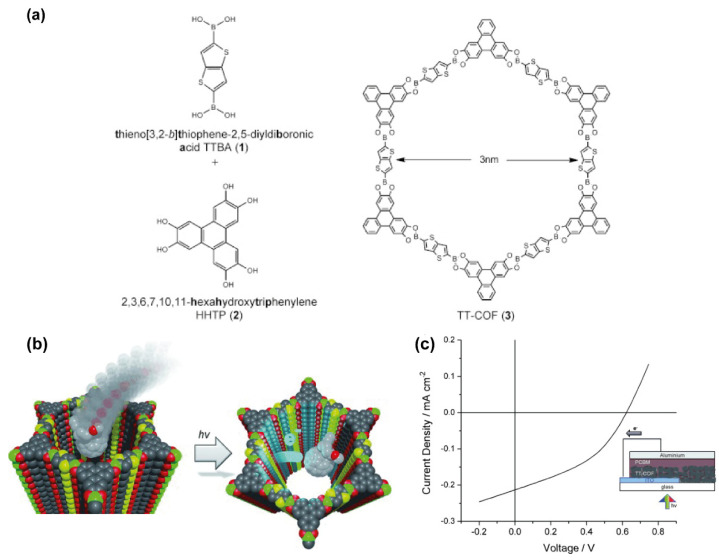
(**a**) Chemical structures of the TTBA and HHTP linkers, and the TT-COF. (**b**) Schematic representation of the inclusion of PCBM in the pores of TT-COF together with the electron transfer happening from the COF to the PCBM upon irradiation with light. (**c**) Graph showing the current vs. voltage behavior of a TT-COF-based photovoltaic cell upon irradiation with a solar light simulator. Reproduced and adapted with permission from [42]. Copyright © 2021 WILEY-VCH Verlag GmbH and Co. KGaA, Weinheim, Germany.

The larger pores created as a result of the formation of stackable thiophene-based COFs from the pilling up of their 2D counterparts enable the inclusion of larger organic compounds. For example, the solvothermal condensation of thieno[3,2-*b*]thiophene-2,5-diyldiboronic acid (TTBA) and the polyol 2,3,6,7,10,11-hexahydroxytriphenylene (HHTP) produced a porous COF (TT-COF, Figure 14a) as a result of the staking of the TTBA building blocks [42]. This TT-COF has a high surface area (1810 m^2^ g^−1^) and a pore size of 3 nm (Figure 14a). Based on those excellent properties, the TT-COF was used as a host for an electron acceptor fullerene derivative, [6,6]-phenyl-C61-butyric acid methyl ester (PCBM, Figure 14b). Considering the high charge-carrier mobilities and stability under ambient conditions of semiconducting polymers based on the thieno[2,3-b]thiophene building blocks [73], along with the demonstrated photoinduced charge transfer from polymerized thienothiophene derivatives to fullerene-based acceptor molecules [74], the PCBM@TT-COF complex was proposed as an ordered bulk heterojunction material for implementation in a photovoltaic device (Figure 14c). Steady-state and time-resolved spectroscopic studies showed charge transfer from the photoconductive TT-COF donor network to the encapsulated PCBM phase in the pore system. To demonstrate the applicability of this system, the authors created a COF-based photovoltaic device by incorporating a COF film of about 200 nm thickness into a ITO/TT-COF:PCBM/Al device structure (Figure 14c). Under simulated AM1.5G full sun illumination, the device yielded open-circuit voltage of 622 mV, a short-circuit current density of 0.213 mAcm^−2^ (Figure 14c), and 40% fill factor, thus giving rise to a power conversion efficiency of 0.053%. In this device, most of the photocurrent was generated from wavelengths below 530 nm, with a maximum external quantum efficiency of 3.4% observed at 405 nm [42].

**Figure 15:**
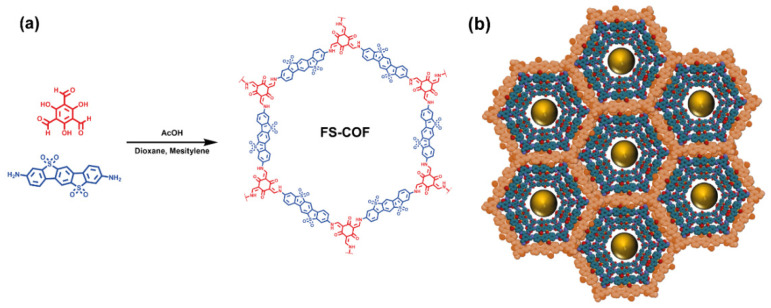
(**a**) Chemical structures of the 1,3,5-triformylphloroglucinol and 3,9-diamino-benzo[1,2-*b*:4,5-*b*′]bis[1]benzothiophene sulfone linkers along with the resulting FS-COF (**b**) Representation of the stacked porous structure of FS-COF.

Another example of a mesoporous thiophene-based COF able to encapsulate different guest molecules is the FS-COF reported by X. Wang and coworkers (Figure 15) [51]. This COF was synthesized by a Schiff-base condensation reaction of 1,3,5-triformylphloroglucinol and 3,9-diamino-benzo[1,2-*b*:4,5-*b*′]bis[1]benzothiophene sulfone (FSA). The authors demonstrated that the FS-COF could behave as a good photocatalyst for the hydrogen evolution reaction. Indeed, they compared the hydrogen evolution rate (HER) of this COF with another two synthesized COFs based on 4,4′′-diamino-p-terphenyl (TP-COF) and 3,7-diaminodibenzo[*b*,*d*]thiophene sulfone (S-COF); and found that the HER of FS-COF (10.1 mmol g^−1^ h^−1^) was ~10 and ~2 times higher to that obtained for the TP-COF (1.6 mmol g^−1^ h^−1^) and S-COF (4.44 mmol g^−1^ h^−1^) [51]. Interestingly, the authors incorporated a wide range of dyes within the mesoporous cavities of the FS-COF intending to enhance the HER. Firstly, they incorporated the 2′,7′-dichlorofluorescein dye, which decreased the photocatalytic activity of the COF, and Rose Bengal (sodium salt of 4,5,6,7-tetrachloro-2′,4′,5′,7′-tetraiodofluorescein), who slightly enhanced its performance. After that, they incarcerated Eosin Y (2′,4′,5′, 7′-tetrabromofluorescein) dye within the FS-COF and observed that the HER was enhanced by a 60% (from 10.1 to 13 mmol g^−1^ h^−1^). However, the absorption of Eosin Y largely overlaps with that of the COF, so the observed HER enhancement may be ascribed to an increase in the total absorption cross-section. In fact, similar increment in the HER value was observed when the amount of COF (without any dye) was augmented. Hence, to induce a real improvement of the HER, the authors encapsulated a red light absorbing dye, **WS5F** ((E)-2-cyano-3-(5-(2-octyl-7-(4-(p-tolyl)-1,2,3,3a,4,8b-hexahydrocyclopenta[b]indol-7-yl)-2H-benzo[d][1,2,3]triazol-4-yl)furan-2-yl)acrylic acid). Upon the encapsulation of this dye, the absorption of the WS5F@FS-COF was broader, spanning up to ~750 nm, and therefore, enhancing the number of photons that this material can absorb. This photon absorption improvement was proposed as the reason behind the astonishing boost of the HER from 10.1 (for COF) to 16.3 mmol g^−1^ h^−1^ (COF + dye). These results highlight the great potential of hybrid materials based on thiophene-COFs incorporating sensitizing compounds in their porous structure.

## 6. Conclusions

Thiophene is a widely used building block of materials for variety of light-driven applications [75,76,77,78]. Although thiophene and its oligomers present many favorable photophysical properties, the resulting supramolecular structures are often limited by inherent disorder and heterogeneous environment, which in turn reduces the ability to fine tune and control their optoelectronic properties. It is, therefore, expected that the incorporation of thiophene-based building units in COF structures could provide intriguing functionalities in the ensuing materials. Tuning the structural and physical properties of a COF while maintaining its porosity and crystallinity can pose significant synthetic challenges, especially in the case of thiophene, which is characterized by geometrical restrictions not observed for other planar aromatic building units [79]. However, as demonstrated in this minireview, several strategies have been developed to overcome these limitations. Beyond controlling the morphological aspects, the photophysical and electronic properties of the thiophene scaffolds can be tuned by modification with the appropriate conjugated spacers (traditionally vinylene or ethynylenes) or appropriate chemical derivatization in the free alfa or beta position of the heterocycle. These additional strategies are not fully exploited yet and should augment the use of thiophene derivatives in the construction of photoactive COFs. These approaches allow for the thiophene-based COF backbones and chemical pore environment to be finely tuned towards the desired structural and optoelectronic properties while preserving the layered arrangement giving rise to 2D and/or stackable ordered porous architectures. The successful implementation of the methods reviewed herein has resulted in advances in the development and application of thiophene-based COFs for photonic, photocatalytic and photosensor applications. On the other hand, there is a significant gap in our understanding of the charge carrier generation, separation, mobility, and diffusion in these complex molecular systems. The charge carrier generation and separation processes usually occur within the first few hundreds of femtoseconds following optical excitation, while the latter process encompasses a wider timescale. Another pending issue that still must be resolved and can significantly affect the potential application of thiophene based COFs in photonics is related with the sample structure and the control of structural defects. Thus, the use of advanced spectroscopy and microscopy techniques such as ultrafast vibrational, X-ray absorption and terahertz spectroscopies, and 4D-electron microscopy could provide a clearer picture on the structure–shape–topology–dynamics relationship of thiophene-based COF systems. These studies can be further supported by theoretical calculations with more efficient methods. This knowledge will guide the design and synthesis of more sophisticated COF materials with precise structural and photophysical properties.
